# The reasons for comparative effectiveness clinical trials of arteriovenous fistula versus graft strategy in older adults on hemodialysis with a catheter 

**DOI:** 10.5414/CN111227

**Published:** 2023-10-25

**Authors:** Mariana Murea, Michael Allon

**Affiliations:** 1 Department of Internal Medicine, Section on Nephrology, Wake Forest School of Medicine, Winston-Salem, NC, and; 2 Division of Nephrology, Department of Medicine, University of Alabama at Birmingham, Birmingham, AL, USA

**Keywords:** arteriovenous access, fistula, graft, hemodialysis, older adults

## Abstract

Clinicians and patients are guided by observational studies to make one of the most consequential decisions for patients with advanced kidney disease: the selection of the “right” hemodialysis vascular access. More than a decade ago, a call for randomized clinical trials was made to equitably compare clinical outcomes between arteriovenous (AV) fistulas (AVFs) and AV grafts (AVGs). Mounting evidence suggests that trade-offs between AVF- and AVG-related outcomes are context dependent. In this article, we summarize four streams of evidence that collectively underpin the burden of equipoise between the two types of AV access in older adults with comorbidities who are on hemodialysis with a central venous catheter.

## Background 

Each year, over 500,000 people in the United States receive life-saving hemodialysis treatments for end-stage kidney disease (ESKD), more than half of whom are adults 60 years of age or older [1]. For these patients, a point of entry into the circulatory compartment – in the form of an arteriovenous (AV) fistula (AVF), AV graft (AVG), or central venous catheter (CVC) – is required to deliver blood to the extracorporeal circuit and dialysis machine [2]. Patients with a CVC are at risk for serious complications, including bacteremia, catheter dysfunction, and central vein stenosis. In observational studies, patient survival was substantially worse in those dialyzing with a CVC, as compared to an AVF or AVG [3, 4, 5, 6], providing further justification for avoiding CVCs. Those findings generated the prevailing paradigm of a surgically created AVF as the preferred vascular access for all patients on hemodialysis [7]. 

Thus, a major goal in clinical practice is to attain a functional AVF or AVG in a timely fashion, to prevent CVC use, or at least limit its duration [8, 9, 10, 11, 12, 13, 14]. Whereas CVCs are available for hemodialysis as soon as they are placed, AVFs and AVGs require several weeks to months before they are suitable for use. Until that happens, patients remain CVC-dependent for receipt of hemodialysis. The preference of AVFs over AVGs (“fistula first”) is primarily based on older studies, enrolling younger patients with few co-morbidities, that reported that AVFs had superior patency for dialysis and required fewer interventions to maintain such patency [15, 16]. More recent observational studies performed in older dialysis populations with higher co-morbidity have questioned the widely accepted superiority of AVFs over AVGs. On the basis of these later studies, a call for randomized clinical trials was made more than a decade ago, to conclusively compare the benefits and risks associated with each type of AV access [17]. The justification of equipoise between AVF- and AVG-related outcomes has only deepened over the past decade, in particular for older adults on hemodialysis with multiple comorbidities. Herein, we summarize four streams of evidence that collectively underscore the large knowledge gap vis-à-vis benefits and risks between AVFs and AVGs in older adults. 

## The AVF-centered practice was built on data from younger population and retrospective studies 

Landmark studies that formed the basis of AVF-centered practice were conducted in the mid-to-late 1990s and were observational in nature; at that time, the mean age of patients treated with hemodialysis was < 60 years [3, 4, 5, 6]. Inherent by design, patients with an access other than AVF were systematically different than those with an AVF, i.e., had more comorbidities and poorer vasculature to maintain the vascular access [3, 4, 5, 6, 18, 19]. 

Over the last decades, more than half of all patients starting dialysis are > 60 years of age [1]. Inherent to older age and advanced kidney disease are multiple comorbidities, polypharmacy, undertreated conditions, and a higher prevalence of physical disabilities [20, 21]. This is a vulnerable subset of the dialysis population who are often disabled and experience treatment burdens and distressing symptoms related to HD. These include, but are not limited to, time spent in dialysis, vascular access complications, and repeat hospitalizations [22]. 

Recent studies have indicated that the choice of vascular access approach and access development is a surrogate marker for severity of comorbidities, which in and of themselves affect clinical outcomes [23, 24]. The decision to place an AVF and the outcome of AVF maturation is a surrogate marker of a healthier clinical status in ways unrecognized even by sophisticated statistical analyses (e.g., perceived better prognosis, less severe comorbidities, observable functional status) [18, 25]. In a large cohort from the U.S. Renal Data System comprised of incident patients ≥ 67 years old, those who initiated hemodialysis with a CVC and had a history of AVF primary failure experienced 34% lower mortality rates than those who initiated hemodialysis with a CVC but never underwent AVF placement – despite both groups receiving hemodialysis via CVC [18]. This observation suggests that patients who receive an AVF are intrinsically healthier than those who do not undergo AVF placement. The association between the initial vascular access type and 5-year mortality was examined in 117,277 hemodialysis patients aged 67 – 90 years using proportional hazard models to account for functional status (i.e., inability to ambulate, inability to transfer, needs assistance with activities of daily living, or institutionalized) and number of hospital days within 2 years prior to dialysis initiation. The association attenuated –23.7% after adjusting for demographics, comorbidities, and pre-dialysis nephrology care; adjustment for health status attenuated the association by another –19.7% (–18.2, –21.3) [19]. The observed attenuation in mortality differences previously attributed to access type alone underscores that the vascular access is much more a proxy of underlying health status than a direct and independent cause of clinical outcomes. To render the patients with AVF and AVG comparable for known and unknown baseline confounders, prospective studies with unbiased patient sampling will more reliably estimate effects of exposure (i.e., type of AV access placed surgically) on outcomes unlikely to be explained by other factors, such as confounding or reverse association. This goal can be accomplished only by well-designed randomized controlled trials. 

## Age-dependent biological factors independently affect vascular access outcomes 

Older adults are particularly susceptible to failure of AVF development [26, 27, 28]. Compared to younger counterparts, older adults have a two-fold higher rate of AVF failure, corresponding to up to a 60% likelihood of failed AVF access surgery [27, 28]. Arterial calcifications, more prevalent in the aging population, hinder AVF maturation [29, 30]. Conversely, vascular calcifications and fibrosis were associated with lower rates of AVG failure [31]. Other biological factors associated with AVF – but not AVG – maturation failure include comorbidities that are extremely common in older patients with ESKD: coronary artery disease, peripheral arterial disease, and diabetes mellitus [27, 32, 33]. A meta-analysis of 13 studies concluded that older patients have a 50 – 65% higher risk of primary AVF failure and an 80% higher risk of secondary AVF failure compared with younger patients [32]. Nevertheless, some observational studies continue to report AVF maturation rates in older adults that are similar to the profile of AVF development in younger counterparts [34]. Other studies suggested no significant difference in AVF maturation and survival rates by age, sex, and presence of diabetes mellitus [35]. Clinical trials that analyze access-related outcomes following AVF vs. AVG creation in a highly pertinent context, i.e., older adults with comorbidities linked with AVF failure, would meet the goal of providing information for patient-tailored clinical decision. 

## The balance of tradeoffs between AVF and AVG is different in older adults compared to their younger counterparts 

Impaired AVF maturation (i.e., the failure of a new AVF to develop into a usable vascular access) leads to prolonged use of CVCs (with attendant risk of sepsis and central venous stenosis) and the need for further invasive access procedures to create a more suitable AV access [36]. On the one hand, AVGs confer shorter intervals (44 vs. 97 days) to successful cannulation than do native AVFs [37, 38, 39]. On the other hand, AVFs, if they mature, might have longer functional lifespans than AVGs [40, 41, 42]. However, when one takes failed AVFs into account (i.e., an intent-to-treat analysis), functional longevity is similar between AVFs and AVGs [42, 43]. Moreover, current clinical practice employs AVG placement in patients who lack vasculature suitable for AVF creation, obscuring objective comparisons in access functionality between each type of AV access. Furthermore, comorbidities associated with AVF maturation failure have not been shown to impact AVG cannulation rate [32, 33, 34]. In some ways, these properties render AVGs a superior “CVC-sparing” strategy than AVFs. In aretrospective cohort of patients ≥ 67 years of age identified between May 2012 and May 2017 in the U.S. Renal Data System, who initiated HD without a permanent access in place and had a first AVF (n = 14,532) or AVG (n = 3,391) placed within 1 year after HD initiation, creation of an AVF was associated with significantly greater cumulative catheter dependence than placement of an AVG (80.1 vs. 54.6 days per person-year) and a lower proportion of catheter-free survival time (78.1 vs. 85.1%) after 3 years of follow-up [45]. The authors concluded that as the long-term benefits in terms of catheter dependence of an AVF are not realized in many elderly patients, specific patient characteristics should be considered when making decisions regarding vascular access [45]. Thus, comparing access-related outcomes in patients with vasculature suitable for surgical creation of either type of AV access would allow for better characterization and comparison of the strengths and weaknesses between AVFs and AVGs. 

## The pathophysiologic mechanism underlying the purported link between the AV access type and dialysis survival is unclear 

Vascular access-related infectious complications were proposed as the primary mechanism responsible for the excess mortality observed with CVCs vs. AVGs and AVFs, but this was refuted in recent cohorts. Quinn et al. [25] showed that only 2.3% of all deaths in patients on hemodialysis are access-related. Furthermore, using mediational analysis, the association between access type and mortality was identical in models including and excluding access complications [4, 25, 46]. This observation suggests that the higher mortality in patients with a CVC or AVGs vs. those with AVFs is not the result of increased rates of access complications with the former vs. the latter type of access. In some studies, older adults exhibited similar access infection rates [47] and survival [37, 48] whether they had AVFs or AVGs. More recent retrospective studies that analyzed the association between the type of vascular access used and older adults’ survival showed either no difference in survival [37, 48] or better survival [49] in those using AVFs vs. AVGs. Nevertheless, a number of contemporary observational studies continue to indicate that AVF is the preferred vascular access of choice in terms of long-term outcomes in patients > 65 years on hemodialysis [49]. An important flaw of retrospective data analyses is the lack of clarity about patient selection criteria and the unclear timing of AV access placement. A clear distinction needs to be made between older adults with comorbidities receiving hemodialysis with a CVC vs. older adults who are in pre-dialysis stages. Thus, by design, a randomized clinical trial would address the effects of selection bias and residual confounders on outcome measurements in ways that observational studies cannot ascertain. 

## Conclusion 

In conclusion, current vascular access practice – centered on surgical AVF creation – is based on 30-year-old retrospective research and observational studies [3, 4, 5, 6]; is not tailored to patient-level characteristics [24, 50, 51]; and has not been evaluated in clinical trials [17]. Each type of AV access has advantages and disadvantages (Table 1), the extent of which can vary with patients’ underlying biological factors [8, 9, 10, 11, 12, 13, 14], rendering the net benefit between AVF and AVG uncertain in older adults with comorbidities. Acknowledging the current evidence gap, the 2019 updated National Kidney Foundation Kidney Disease Outcomes Quality Initiative (NKF KDOQI) Clinical Practice Guideline for Vascular Access recommends placement of the “right access, in the right patient, at the right time, for the right reasons” and calls for clinical trials to determine the benefits of AVF vs. AVG access approach in older adults [52]. Obtaining comparative results between alternative AV access strategies through rigorously performed randomized studies would meet a critical need. To address this evidence gap, we designed and launched a clinical trial to compare the effectiveness and safety of AVF vs. AVG access strategy in older adults (NCT04646226), with the goal of providing robust scientific evidence for clinicians and patients to make optimally-informed decisions about hemodialysis vascular access choice [53]. More investigations in the vascular access field are desperately needed to close knowledge gaps and improve the care of patients on hemodialysis. 

## Funding 

The authors are sponsored by the National Institutes of Health (NIH)/National Institute on Aging (NIA), R01AG071803. 

## Conflict of interest 

The authors declare no conflict of interest. 

**Table 1.** Arteriovenous (AV) fistula (AVF) vs. AV graft (AVG): advantages and disadvantages*.

AVF	AVG
Advantages	
↑ Functional longevity^#^ ↓ Thrombosis rate ↓ Infection rate^#^	↑ Maturation rate ↓ Interventions to aid maturation ↓ Lag-time creation to cannulation ↑ Surface area for cannulation Technically easy to cannulate Easier to repair
Disadvantages	
↑ Failure to mature ↑ Catheter dependence ↑ Interventions to aid maturation	↑ Thrombosis rate ↓ Functional longevity^# ^ ↑ Infection rate

*Based on current retrospective, observational data. ^#^Properties controverted in older adults. ↑ increased (higher rate); ↓ decreased (lower rate).
